# Sensing the Worst: Neurophenomenological Perspectives on Neutral Stimuli Misperception in Schizophrenia Spectrum

**DOI:** 10.3389/fnhum.2017.00269

**Published:** 2017-06-02

**Authors:** Mariateresa Sestito, Josef Parnas, Carlo Maggini, Vittorio Gallese

**Affiliations:** ^1^Unit of Physiology, Department of Neuroscience, University of ParmaParma, Italy; ^2^Department of Psychology, Wright State UniversityDayton, OH, United States; ^3^Psychiatric Center Hvidovre, University Hospital of CopenhagenCopenhagen, Denmark; ^4^Center for Subjectivity Research, University of CopenhagenCopenhagen, Denmark; ^5^Psychiatric Division, Department of Neuroscience, University of ParmaParma, Italy; ^6^Institute of Philosophy, School of Advanced Study, University of LondonLondon, United Kingdom

**Keywords:** aberrant salience, affect recognition, delusions, hyper-reflexivity, emotion, neutral stimuli misperception, phenomenology, schizophrenia spectrum

## Abstract

While investigating social cognitive impairments in schizophrenia, prominent evidence has been found that patients with schizophrenia show a tendency to misclassify neutral stimuli as negatively valenced. Within this population, patients presenting delusions are more prone to this phenomenon. In a previous study, Schizophrenia spectrum (SzSp) patients rated positive, negative and neutral stimuli that were multimodally presented, while assessed with a checklist exploring anomalous subjective experiences and evaluated for positive and negative symptomatology. In the present work, we aimed to further explore the relationship between neutral stimuli misperception, anomalous experiences and positive/negative symptoms in SzSp patients. To this end, we adopted a dimensional approach by reconstructing from available data: (1) four *a priori* scales representing essential dimensions of SzSp experiential pathology following Parnas et al. (2005); and (2) five clinically meaningful factors to describe illness severity derived by Toomey et al. (1997). Results showed that although overall patients correctly recognized the target emotions, those who misinterpreted neutral auditory cues as negatively valenced also presented higher scores in Perplexity (PY), Bizarre Delusions (BD) and Disorganization (Di) dimensions. Moreover, a positive association between BD and both PY and Self-Disorder (SD) dimensions emerged, suggesting that psychotic symptoms may be directly linked to patients’ subjectivity. In an attempt to comprehensively capture the multilayered neutral stimuli misperception phenomenon in SzSp, we aimed at bridging phenomenology and neurobiology by connecting the levels of molecular neurochemistry (i.e., altered dopaminergic neurotransmission), system neuroscience (aberrant salience of perceptual details) and psychopathology (the chain involving hyper-reflexivity, self-disorders and the emergence of delusions).

## Introduction

Identifying neurobiologically rooted impairments in cognition has become an increasingly reliable way to detect endophenotypes of core components of schizophrenia. In the matter in question, disturbances in perhaps the most broadly studied domain of social cognition—emotion perception—held the promise of being a possible candidate as an early sign of the disease (Kee et al., 2004; Leppänen et al., 2008; Eack et al., 2010).

Previous research investigated the presence of social cognitive impairments in facial emotion recognition among patients with schizophrenia and populations at ultra-high risk for psychosis (Schneider et al., 2006; Sestito et al., 2013). The results reported that alongside a preserved sensitivity to detect the target emotions, they were more likely to overattribute emotions to neutral faces, predominantly misinterpreting such faces as negatively valenced (Kohler et al., 2003; Eack et al., 2010; van Rijn et al., 2011; Amminger et al., 2012a,b).

Biological theories of psychosis have accounted for the tendency to misinterpret benign or ambiguous social cues. In psychosis, increased dopamine is observed in the mesolimbic pathway, with dopamine being a key neurochemical determinant of the significance of environmental cues to human motivations. Abnormal increases in this neurotransmitter are proposed to influence the perceived salience of such environmental signs, leading to their *aberrant assignment of salience* (Kapur, 2003). This mechanism is thought to mediate the tendency to interpret neutral faces as emotionally meaningful (Kapur, 2003). Such negative misattribution bias has been shown to be a special trait of those individuals presenting positive symptoms (Holt et al., 2006; Eack et al., 2010). Also, the negative misattribution bias has been found to be more pronounced in patients with longer illness duration, indicating that while deficits are already present at early stages, they seem to progress along a chronic course (Habel et al., 2010). Prominent models of delusion formation further suggest that individuals with persecutory delusions and paranoia are more prone to mis-assigning emotional meaning to neutral information (Bentall et al., 2001). Recent findings showed elevated dopamine synthesis even in prodromal individuals, which is correlated with psychotic symptoms severity (Howes et al., 2009) and predicts later psychotic disorder transition (Howes et al., 2011; Allott et al., 2014).

Empirical evidence has been available since the dawn of schizophrenia research, suggesting that the onset of the illness may be predated or accompanied by characteristic qualitative changes of subjective experience (Berze, 1914; Berze and Gruhle, 1929; McGhie and Chapman, 1961; Huber, 1983; Clerambault, 1992; Janet, 1993). Such not-yet-psychotic—i.e., non-delusional, non-hallucinatory—manifestations may entail various forms of anomalies in the domains of perception, cognition and attention, body and movement awareness, as well as alarming alterations in the domain of self-awareness (Gross, 1989; Sass and Parnas, 2003; Schultze-Lutter, 2009). These anomalies are linked to profound alterations of self-experience such as impaired identity and demarcation, solipsistic detachment from common sense attunement to the world, and defective temporalization (Bovet and Parnas, 1993). According to Huber (1983), these so-called *basic symptoms* constitute an intermediate—i.e., transphenomenal—level between the basic biological processes and overt psychotic symptoms. These experiential anomalies indeed have been shown to aggregate selectively in patients with schizophrenia spectrum (SzSp), suggesting a basic phenomenological affinity of these disorders (Parnas et al., 2005; Nordgaard and Parnas, 2014). Basic symptoms in a more pragmatic clinical context may be potentially effective for early differential diagnosis (Klosterkötter et al., 2001; Parnas and Handest, 2003).

Within this framework, little is known about the degree to which the negative recognition bias is associated with manifestations of SzSp anomalous subjective experiences and their possible relationship with positive and negative symptoms. With the rationale to begin to address this matter, we considered behavioral and clinical data gathered from a previous study (Sestito et al., 2015), whereby SzSp patients were tested with a multimodal paradigm in order to investigate affect recognition. By embracing a *phenomenological leverage* (Nordgaard et al., 2008) in conceiving full-blown signs of schizophrenia, we intended to conduct an exploratory analysis in order to investigate whether possible connections are detectable between patients’ psychotic symptoms and experiential dimensions. We considered such supplementary data acquired following this protocol to provide a useful way to begin to approach this inquiry, as patients were assessed with a checklist exploring anomalous subjective experiences and evaluated for positive and negative symptomatology.

## Materials and Methods

### Participants

Nineteen outpatients (14 males, 5 females, mean age 34.11 years; SD ±6.73) were recruited at the Psychiatry Section of Parma University Department of Neuroscience. All of them were diagnosed with a Schizophrenia Spectrum (SzSp) disorder (i.e., schizophrenia (*N* = 15) or schizotypal personality disorder (*N* = 4) according to DSM-IV diagnostic criteria (American Psychiatric Association, 1994)) and were clinically stable at the time of the assessment. This clinical sample has been considered in a parallel study focusing on different inquiries than the one currently at stake (Sestito et al., 2015).

Patients suffering from organic brain disorders, brain injury, alcohol or substance abuse and mental retardation were excluded from the study. The Scales for the Assessment of Positive and Negative Symptoms (SAPS; SANS; Andreasen, 1984a,b) were coded and served as global measures of severity of the disorder. Disturbances of subjective experience were explored through the Italian version of the Bonn Scale for the Assessment of Basic Symptoms (BSABS; Gross et al., 1992). The BSABS interviews were conducted by a senior psychiatrist (CM) with extensive research interview experience and principal translator of BSABS into Italian. Each patient was assessed in a semi-structured way about the anomalies of experience on a lifetime basis. A total amount of 103 items were rated for presence/absence (98 principal items plus five items exploring coping strategies).

Patients were all under psychopharmacologic treatment with antipsychotics, hence the cumulative measure of lifetime drug exposure was calculated following Andreasen et al. (2010). Demographic and psychopathological features of the sample are reported in Table 1.

**Table 1:** **Demographic variables and psychopathological features of the Schizophrenia Spectrum (SzSp) sample and its constituent subgroups (Schizophrenia and Schizotypal personality disorder)**.

		SzSp sample (*N* = 19)			Schizophrenia subgroup (*N* = 15)			Schizotypal personality disorder subgroup (*N* = 4)		
	Mean	SD	Range (scale range)	Mean	SD	Range (scale range)	Mean	SD	Range (scale range)
Age (years)	34.11	6.73	25–49	32.180	6.55	25–49	39.00	5.60	32–44
Gender, F/M	5/14			5/14			0/4		
SAPS	24.01	16.26	1–58 (0–170)	26.61	16.17	1–58 (0–170)	14.25	14.36	2–35 (0–170)
SANS	46.34	17.63	15–83 (0–125)	48.16	17.64	17–83 (0–125)	39.5	18.27	15–58 (0–125)
BSABS	41.72	16.52	29–86 (0–103)	42.05	17.97	29–86 (0–103)	40.50	11.36	29–53 (0–103)
Length of illness (years)	11.06	4.84	2–24	11.21	5.03	2–24	10.50	4.65	6–17
Age at first recognized psychotic episode	24.06	4.30	19–34	22.87	2.59	19–28	28.5	6.81	20–34
Number of hospitalizations	3.35	1.82	0–7	3.78	1.47	2–7	1.75	2.36	0–5
Dose of typical and atypical antipsychotics^a^	26.41	19.54		31.13	19.32		8.71	4.52		
Dose of atypical antipsychotics^a^	20.01	15.64		23.51	15.67		6.86	5.59		
Dose of typical antipsychotics^a^	6.40	5.72		7.62	5.79		1.85	2.20		

*^a^Drugs are expressed as the cumulative value measured in dose-years in the form of (chlorpromazine equivalent in mg) × (time on dose measured in years; Andreasen et al., 2010)*.

All participants gave their written informed consent before entering the study, which was approved by the Ethics Committee of the University of Parma and carried out according with the ethical standards of the 2013 Declaration of Helsinki.

### Experimental Paradigm: Stimuli and Procedure

**Figure 1:**
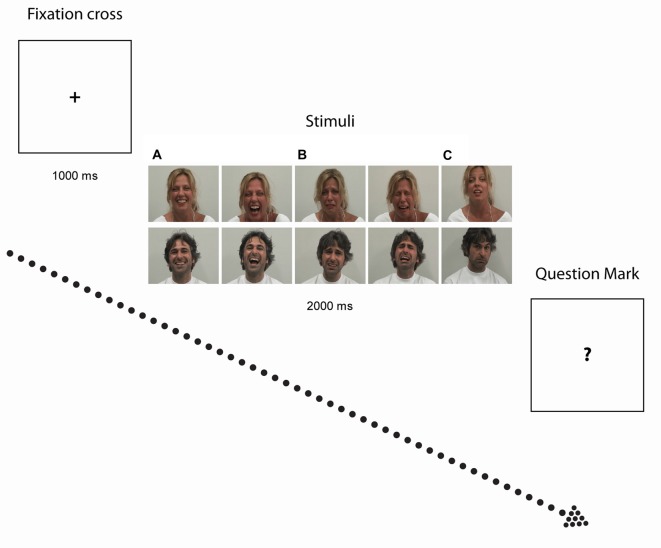
**Experimental paradigm**. Photographs illustrate examples of stimuli depicting Laugh **(A)** Cry **(B)** and Neutral **(C)** stimuli. After exposure to each video clip, when the question mark symbol (“?”) appeared on the screen, participants had to quantitatively rate the emotional value of the stimuli.

The experimental paradigm herein used (Sestito et al., 2013) followed the subsequent procedure. Participants were presented with 2-s color video clips portraying two actors displaying positive (laugh), negative (cry) and neutral (control) facial expressions and sounds in visual (i.e., Video) and auditory (i.e., Audio) modalities. Video or Audio modalities were either in isolation (i.e., V or A alone), or combined (i.e., AV). AV combination were either congruent (Audio-Visual Congruent, AVC) i.e., A and V conveying the same emotion (e.g., Laugh) or incongruent (Audio-Visual Incongruent, AVI), i.e., A and V conveying contradictory information (for example, in AVI Cry, participants saw an actor laughing but heard crying, whereas in AVI Laugh, participants saw an actor crying but heard laughing). The neutral video clips showed actors making various faces associated with specific vocalizations that did not imply any particular emotional content. The sound associated to such stimuli were vocalizations similar to “ahh”, “ohh”, or “ehmm”. Participants were asked to quantify the emotional value of the stimuli (see Figure 1) by verbally rating their intensity on a 7-point Likert scale ranging from −3 (very negative) to +3 (very positive), whereby 0 indicated lack of perceived emotional content. The validation of stimuli and experimental procedure employed have been reported in detail elsewhere (Sestito et al., 2013).

### Data Analysis

#### Data Reduction

Participants’ behavioral rating scores were analyzed following the same procedure defined in the validation protocol (Sestito et al., 2013). Following the specific aim of this study, only behavioral rates given to neutral stimuli have been taken into account, for those modalities which turned out to be informative as established statistically (see below).

In order to explore possible relations between behavioral evidence and phenomenological experiences believed to reflect a distinctive phenotype of the schizophrenia psychopathology, four *a priori* scales were constructed from the BSABS items following Parnas and colleagues (Parnas and Handest, 2003; Parnas et al., 2005). BSABS items were grouped into four rational scales representing essential dimensions of SzSp experiential pathology: (1) *Perplexity* (PY), (2) *Perceptual Disorders* (PD), (3) *Self-Disorder* (SD) and (4) *Cenesthesias* (CEN). We herein choose to follow such a scale conformation adopted by Parnas et al. (2005) for many reasons. First, evidence has been previously provided demonstrating that individuals with schizophrenia and schizotypal disorders scored equally on such subjective dimensions (Parnas et al., 2005). Moreover, the SD scale here considered comprises some items usually considered to be “cognitive” (e.g., thought block and interference), in line with the view considering such anomalies of thinking as a facet of SD (Parnas and Handest, 2003).

Finally, scores derived from SANS and SAPS were arranged following Toomey et al. (1997). These Authors constructed, at item level, some clinically meaningful dimensions able to describe illness severity in a more informative way than the global scores themselves: (1) *Diminished Expression* (DE), (2) *Disorganization* (Di), (3) *Disordered Relating* (DR), (4) *Bizarre Delusions* (BD) and (5) *Auditory Hallucinations* (AH).

To ensure a good internal consistency, all scales were subjected to an item analysis, intended to maximize alpha coefficient (Cronbach, 1951). Only the scales reaching a satisfactory internal consistency (*α* > 0.50) were retained in the subsequent analyses.

#### Statistical Analyses

First, normality of all variables was evaluated through visual inspection of histograms and the application of the Kolmogorov-Smirnov test. It turned out that assumptions for applying parametric tests were met for all variables.

The rating scores of each participant were averaged on the basis of modality and emotion and entered into a 4 (Modality: AVC, AVI, Audio, Video) × 3 (Emotion: Laugh, Cry, Control) repeated measures ANOVA, with Modality and Emotion as within-participants factors.

In checking for the assumptions for running the regression analysis, a preliminary check of the correlation matrix was done and those variables that showed a strong linear association (0.85 was used as cutoff) were not considered in the subsequent analysis.

A hierarchical regression analysis (forward stepping) was then conducted in order to determine the variance explained in the dependent variables (i.e., behavioral ratings), with Parnas’ and Toomey’s scales as predictors. As the sex variable was not balanced in our sample, it was included among predictors.

For all performed analyses, *p* < 0.05 was considered to be statistically significant.

## Results

**Figure 2:**
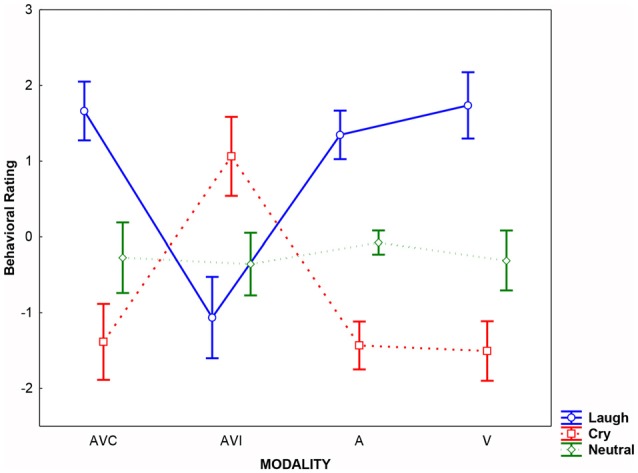
**Averaged rating scores detected for each modality (AVC: Audio-Video Congruent, AVI: Audio-Video Incongruent; A: Audio, V: Video) and emotion (Laugh, Cry, Neutral)**. Error bars represent the standard deviation (SD).

Results of the analysis performed on behavioral rating scores showed that the Emotion factor was significant (*F*_(2,36)_ = 60.58, *p* < 0.0001; $\eta_{\text{p}}^{2}$ = 0.66). *Post hoc* comparisons (Bonferroni corrected for multiple comparisons) revealed that Cry was rated by SzSp participants more negatively than Laugh, and Neutral stimuli were considered as devoid of any emotional content (Laugh vs. Cry; Neutral vs. Cry and Laugh all *p*_s_ < 0.004). Moreover, the Modality × Emotion interaction was significant (*F*_(6,108)_ = 55.64, *p* < 0.0001 $\eta_{\text{p}}^{2}$ = 0.76), meaning that during AVI modality, SzSp participants based their ratings following the visual content of the stimuli—that is, cry in AVI Laugh condition (in which participants saw crying and heard laughing) and laugh in AVI Cry condition (in which participants saw laughing and heard crying; AVI Laugh vs. other modalities all *p*_s_ < 0.0001; AVI Cry vs. other modalities all *p*_s_ < 0.0001; Figure 2).

After a correlation matrix inspection (see Table A in the Supplementary Material Appendix), a strong linear correlation among behavioral ratings given in the AVC, AVI and V modalities emerged (AVC vs. AVI, V *p*_s_ > 0.88; AVI vs. AVC, V *p*_s_ > 0.89; V vs. AVC, AVI *p*_s_ > 0.88) so that they were excluded from the subsequent analyses. Finally, only the condition Audio Neutral (A Neutral) was retained as dependent variable, hence entered in the regression analysis.

After item analyses, all Toomey’s scales and three out of the original four Parnas’ *a priori* scales, i.e., (1) *Perplexity* (PY), (2) *Self-Disorders* (SD), (3) *Cenesthesias* (CEN) reached a satisfactorily internal consistency (alpha value ≥0.50) and were considered in the following analyses (for item composition and *alpha* coefficients for each scale, see Tables B and C in the Supplementary Material Appendix).

**Figure 3:**
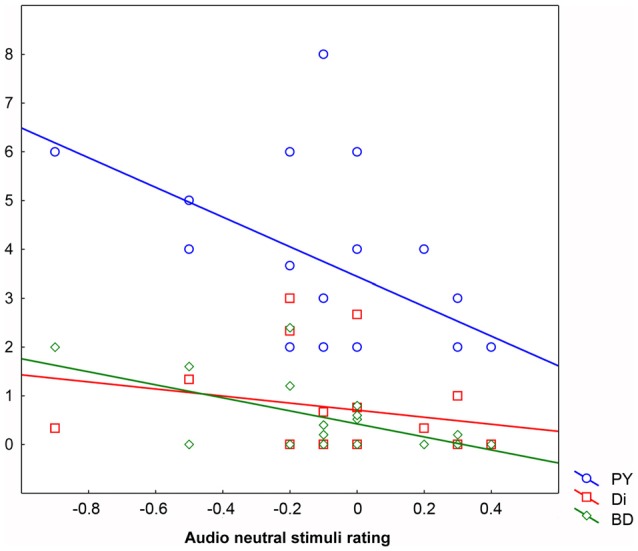
**Behavioral ratings in the Audio Neutral condition (*X* axis) as explained by scores obtained in the three predictors Perplexity (PY; *Y* axis), Disorganization (Di; *Y* axis) and Bizarre Delusions (BD; *Y* axis)**. Behavioral ratings given below “0” mean that stimuli are rated as negative, ratings given above “0” mean that stimuli are rated as positive, whereas “0” means lack of perceived emotional content.

The hierarchical regression analysis (forward stepping) demonstrated that the behavioral rating given in the A Neutral condition was explained by the combination of four predictors: Bizarre Delusion (BD; *t* = −1.85 β = −0.40, *p* < 0.09 explaining the 24.23% of the variance), Perplexity (PY; *t* = −1.62 β = −0.35, *p* < 0.2 explaining the 21.82% of the variance), Sex (*t* = −1.10 β = −0.21, *p* < 0.30 explaining the 3.66% of the variance) and Disorganization (Di; *t* = −1.00 β = −0.19, *p* < 0.4 explaining the 0.58% of the variance) for which the overall regression model (*F*_(4,14)_ = 3.52, *p* < 0.04, *R* = 0.71, *R*^2^ = 0.50) accounted for 50.17% of the variance. That is, the more participants rated neutral stimuli as negative, the higher the scores in Bizarre Delusion, Perplexity and Disorganization dimensions. Also the inclusion of Sex in the final model indicates males to be more prone to attribute a negative valence to neutral auditory stimuli. No other predictors were included in the regression model (Figure 3).

**Figure 4:**
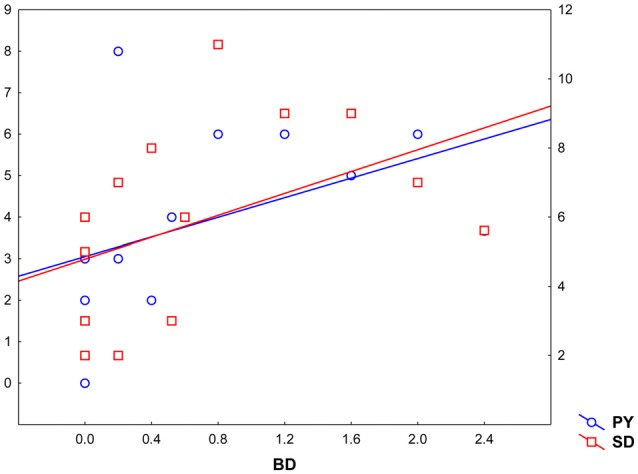
**Correlation between Toomey et al. (1997) Bizarre Delusions dimension (BD, *X* axis) and Parnas and colleagues’ Perplexity and Self-Disorder scales (Perplexity, PY; *Y* left axis; Self-Disorder, SD; *Y* right axis)**.

Pearsons’ correlations between Parnas’ and Toomey’s scales disclosed a positive correlation between Bizarre Delusion (BD) and both Perplexity (PY; *r*_(19)_ = 0.46, *p* < 0.05) and Self-Disorder (SD; *r*_(19)_ = 0.47, *p* < 0.05) subscales (see Table 2 and Figure 4). Given the exploratory nature of this study and the small sample size, we did not correct such correlations for multiple comparisons. Hence, the latter findings should be considered as suggestive of their generalizability to the general schizophrenia population.

**Table 2:** **Correlation matrix contrasting Parnas et al. (2005) scales (PY, Perplexity; SD, Self-Disorder; CEN, Cenesthesias) and Toomey et al. (1997) scales (DE, Diminished Expression; Di, Disorganization; DR, Disordered Relating; BD, Bizarre Delusions; AH, Auditory Hallucinations)**.

	PY	SD	CEN
**DE**	−0.142	0.140	−0.188
	*p* = 0.561	*p* = 0.566	*p* = 0.442
**Di**	0.074	0.008	−0.241
	*p* = 0.763	*p* = 0.976	*p* = 0.320
**DR**	−0.068	−0.071	0.013
	*p* = 0.784	*p* = 0.772	*p* = 0.959
**BD**	**0.461**	**0.466**	0.096
	*p* = 0.047*	*p* = 0.044*	*p* = 0.696
**AH**	−0.138	0.026	0.173
	*p* = 0.572	*p* = 0.914	*p* = 0.479

*p < 0.05. Data in bold are statistically significant.

## Discussion

In this exploratory study, by assuming a dimensional approach to the measurement of experiential anomalies and symptom severity, we aimed at furthering the relationship between neutral stimuli perception, anomalous subjective experiences and positive/negative symptoms in schizophrenia spectrum patients.

Notably, the prior correlation analysis carried out on behavioral ratings given in different modalities disclosed the auditory stimuli to be the most informative likely for their more equivocal nature, thus suitable for studying the ambiguous stimuli misperception phenomenon. The results showed that although overall patients correctly recognized positive, negative and neutral stimuli as such as previously reported (Sestito et al., 2013), those who misinterpreted neutral auditory cues as negatively valenced also presented higher scores in Perplexity (PY; Parnas et al., 2005), Bizarre Delusions (BD; Parnas et al., 2005) and Disorganization (Di; Toomey et al., 1997) dimensions. Also, the inclusion of sex in the final model indicates males to be more prone to this phenomenon. This is not surprising, as sex-related differences in the clinical expression and outcome of schizophrenia have long been recognized (Seeman, 1982). Males are reported to have an earlier onset of the disorder, more severe symptoms, prolonged period of untreated illness and poorer outcome with respect to females (Tandon et al., 2009; Cocchi et al., 2014). Multiple regression analysis demonstrated that the combination of these factors significantly accounted for the magnitude of the negative response bias given in the neutral auditory condition. It should be noted that the Auditory Hallucination (AH) dimension has not been included in the final regression model accounting for ratings given in the neutral auditory condition, confirming that neutral stimuli misreading doesn’t herein merely characterize those patients presenting AH.

Further correlation analyses performed between experiential features (Parnas’ *a priori* scales) and disease severity (Toomey’s scales) showed a significant positive correlation between BD and both PY and SD dimensions in these individuals, suggesting that positive symptoms may be directly linked with patients’ subjectivity.

The data herein reported are in concurrence with previous investigations employing similar emotion recognition paradigms, showing that patients with chronic schizophrenia presenting positive symptoms may also exhibit negative interpretations of neutral stimuli (Bentall et al., 2001; Holt et al., 2006; Eack et al., 2010). Delusions might stem from the misattribution of affective meaning to neutral or ambiguous information—an “affective misattribution bias”. Our findings are consistent with an inappropriate activation of a *salience detector* (Kapur, 2003), as there is certainly enduring support for dopamine dysregulation as a final common pathway in psychosis, described as the *wind of the psychotic fire* (Laruelle et al., 1999). This might lead to the mis-assignment of emotional salience to ambiguous stimuli in the real world and ultimately, to the formation and maintenance of delusions.

Phenomenological psychiatry locates the disturbance of subjective experience in schizophrenia at the level of the pre-reflective and practical immersion of the self in the world, where the commonsense tie to natural reality is formed. Phenomenology describes this as the pre-conceptual intentional self-world relation (Blankenburg, 1971; Bovet and Parnas, 1993).

PY and SD dimensions jointly reflect a structural transformation of the *intentional arch* (Minkowski, 1927)—that is, the most basic relation between the self and the world. The PY scale signifies, in phenomenological terms, a difficulty in seeing the world as a familiar Gestalt, a difficulty in a natural grasp of meaning and hyper-reflexivity. Patients tend to perceive themselves or aspects of the environment as objects of intense reflection, preventing a smooth engagement in the interactions with the world. Isolated aspects of the environment, objects and situations, acquire an intrusive experiential quality, which indeterminately increase their significance. Such objects and situations may be experienced with enhanced emotional meaning. Included among PY scale items, (resulting) derealization (C.2.11 item) implies a change in the experience of the environment: the surrounding world appears somehow transformed, unreal, and strange. There is an increase or accentuation of the physiognomy (Gestalt meaning) of reality and of its isolated aspects, often occurring together with a captivation by details of perception (C.2.9 item) and de-automatization of common every day actions (C.3.3 item). Coherently with our results, derealization may be accompanied by more specific changes of perception, e.g., change in the quality of perceived sounds (Parnas et al., 2005).

The suggestion that the early stage of schizophrenia could be characterized by a breakdown of Gestalt perception was prominent in the work of Matussek (1952) and Conrad (1958). Matussek (1952) described a patient who reported no appreciation of the whole—he only saw details against a meaningless background (p. 92). Parnas et al. (1996) later defined this phenomenon as *impaired perceptual binding capacity*. Arieti (1962) reported in this regard, a patient who “*(‥) could not look at the whole door. She could only look at the knob or some corner of the door. The wall was fragmented into parts*”. Following a loosening of the perceptual context, attention may be captured by incidental details of the environment. Normally, such an aspect of the situation would not reach awareness; its detection however might prompt a search for reasons for its occurrence, which may take a delusional form. Insofar as people normally engage in causal reasoning to make sense of the world, an inappropriate, delusional frame of reference, may provide new elaborative contexts to understand the unexplained dislocated, overtly salient perceptual fragments. Notably on a clinical level, the most consistent clinical correlates of impaired perceptual organization in schizophrenia are the disorganized symptoms (e.g., thought disorders), found to be among the predictors interacting with BD and PY for neutral stimuli misperception in our study.

Notably, the above reported phenomenological descriptions characterizing the hyper-reflective status fit well with the aberrant salience hypothesis for delusions formation (Nelson et al., 2014), conferring an extraordinary richness in terms of experiential correlates upon Kapur’s (2003) model. A comprehensive, plausible picture may thus be drawn by converging evidence related to phenomenology (PY) and psychotic symptoms (BD and Di) in explaining neutral stimuli misperception in SzSp.

As a second result (to be taken with precautions), a correlation has been found among BD and both PY and SD dimensions. The SD scale targets experiences in which the pre-reflective directedness toward the world in unity with the self, which is given prior to any specific act of reflection, becomes shattered and unstable. Under normal conditions, experience and self are not two distinct entities; rather the first person perspective is a medium through which the experience manifests itself (Parnas, 2000). Hyper-reflexivity entails a constant self-monitoring attitude whereby things that are matter of intense reflection are typically treated as “objects”. This attitude creates a pervasive distance between self and experience. And when the self-experience bound becomes loose, then coherence breaks down, leading the delusional versions of the self, divorced from reality, to emerge. Notably in a previous study (Parnas et al., 2005), PY and SD turned out to be the scales that discriminated strongest between the SzSp and non-spectrum, a result confirming the diagnostic importance of such aberrations in the context of Schizophrenia.

Overall, these findings support some specificity of the negative misattribution bias to a combination of experiential features and positive symptoms, whose complex interplay and causality could be herein barely grasped given the exploratory nature of the study. This study indeed, is just a first attempt to comprehensively capture the multilayered turn of events that might characterize neutral stimuli misperception in schizophrenia. A possible movement from the levels of molecular neurochemistry (i.e., altered dopaminergic neurotransmission) to system neuroscience (aberrant salience of perceptual details and neutral cues) to psychopathology (the chain involving hyper-reflexivity, self-detachment and resulting delusional framing of isolated features to make sense of changed reality) may be herein tentatively postulated (Mishara and Fusar-Poli, 2013). Possible arguments aimed at bridging the phenomenological and neurobiological levels may hence be put forward and taken into account as a prompt for possible to-be-planned *ad hoc* studies on this issue, aimed at establishing the contribution of each psychopathological aspect considered.

In conclusion, this research calls for the need to adopt a more refined, emerging approach linking phenomenology, cognitive neuroscience and psychopathology. Such an effort would provide a burgeoning turf for mutual enrichment, and unique insights into vulnerability markers of psychosis (Mishara et al., 1998). Phenomenological accounts and their derived phenotypes can indeed provide the missing link in the chain between genetic or acquired biological vulnerability, the social environment, and the expression of individual positive symptoms. A complex interaction between experiential and full-blown psychotic symptoms might account for emerging problems in reading benign, emotionally un-laden cues adequately. These changes in processing neutral stimuli—primarily triggered by biologically-driven aberrant assignment of salience of perceptual details—seem to embody a peculiar experiential corollary accompanying psychotic symptoms, characterized by hyper-reflexivity and self-detachment.

Notwithstanding the exploratory nature of this study and its intrinsic limitations (the relatively small sample solely including chronic patients), we believe that these findings add important information in research on emotion processing disturbances reflecting possible trait markers of susceptibility to the disorder. However, given the relatively small sample size and the number of relevant variables taken into account, the results here reported should be interpreted with caution and further replication is needed.

Subsequent investigations would endeavor to elucidate the predictive strength of variegated psychopathological factors involved in negative emotion recognition bias for transition into full-fledged psychosis. Clinically, we hereby stress the need to integrate a phenomenological standpoint in the assessment of first-rank symptoms, as only such an approach may allow to grasp their organizing Gestalt (i.e., the altered consciousness of the patient) through the diagnostic process. Research from its side may integrate the rich phenomenological framework into its practice, creating a prolific field to formulate new, experimentally-testable empirical hypotheses strictly tied to patients’ experiential dimension. Continued exploration of these deficits in high-risk populations employing longitudinal designs will be beneficial in the future investigations, thereby pointing to promising directions for early intervention and prevention programs for altering the deteriorative course of the disease.

## Author Contributions

MS: intellectual conceptualization, data collection and analyses, interpretation of data and manuscript drafting. JP: intellectual conceptualization. CM: intellectual conceptualization, data collection, interpretation of data. VG: intellectual conceptualization and interpretation of data. All the authors contributed to the final revision of the manuscript.

## Conflict of Interest Statement

The authors declare that the research was conducted in the absence of any commercial or financial relationships that could be construed as a potential conflict of interest.
