# Supplementary material for: Sensing the Worst: Neurophenomenological Perspectives on Neutral Stimuli Misperception in Schizophrenia Spectrum
Source: Front Hum Neurosci. 2017 Jun 2;11:269. doi: 10.3389/fnhum.2017.00269 (PMC5454073; doi:10.3389/fnhum.2017.00269)
Supplement: Supplementary file 1 [file Data_Sheet_1.docx]

**APPENDIX A**

Correlation matrix of the averaged rating scores detected in the neutral condition for each modality (AVC: Audio-Video Congruent, AVI: Audio-Video Incongruent; A: Audio, V: Video).

|  | **AVC** | **AVI** | **A** | **V** |
| --- | --- | --- | --- | --- |
| **AVC** |  | .894 | -.120 | .884 |
|  |  | p=.000** | p=.624 | p=.000** |
| **AVI** | .893 |  | -.070 | .908 |
|  | p=.000** |  | p=.777 | p=.000** |
| **A** | -.120 | -.0670 |  | .180 |
|  | p=.624 | p=.777 |  | p=.463 |
| **V** | .884 | .908 | .180 |  |
|  | p=.000** | p=.000** | p=.463 |  |

** p < 0.001**APPENDIX B**

Parnas and colleagues’ *a priori* scales (2005) finally retained with Cronbach’s *alpha* coefficients and their item composition.

| **Parnas et al. 2005 *a priori* scales** | | |
| --- | --- | --- |
| *Perplexity a = 0.585* | Hyper-reflexivity  Disturbance in grasping the significance of the world  Captivation of attention by a perceptual detail  Derealisation  Des-automatisation of movements | B.3  C.2.7  C.2.9  C.2.11  C.3.3 |
| *Self-Disorder a = 0.673* | Pervasive sense of lacking vitality  Psychic Depersonalisation  Thought interference  Thought pressure  Thought block  Difficulty of verbal self-expression  Disturbance of thought initiative or intentionality  Mirror-related phenomena (Spiegelphänomen)  Disturbed awareness of continuity of own actions  Physical depersonalisation | A.3  B.3.4  C.1.1  C.1.3  C.1.4  C.1.7  C.1.13  C.2.3  C.2.10  D.1.1 |
| *Cenesthesias a = 0.826* | Migrating sensations  Electric sensations  Thermal sensations (heat or coldness)  Sensations of movement, pulling or pressure inside the body or on its surface  Sensations of abnormal heaviness, lightness or emptiness, of falling or sinking, levitation or elevation  Sensations of extension, diminution, shrinking, enlargement or constriction  Kinesthetic sensations  Vestibular sensations | D.4  D.5  D.6  D.7  D.8  D.9  D.10  D.11 |

**APPENDIX C**

Toomey and colleagues’ scales (1997) with Cronbach’s *alpha* coefficients and their item composition (SANS: Scale for the Assessment of Negative Symptoms; SAPS: Scale for the Assessment of Positive Symptoms).

| **Toomey et al. 1997 scales** | | |
| --- | --- | --- |
| *Diminished Expression a = 0.725* | Unchanging facial expression  Paucity of expressive gestures  Lack of vocal inflections  Decreased spontaneous movements  Affective nonresponsivity  Poor eye contact  Poverty of speech | SANS  SANS  SANS  SANS  SANS  SANS  SANS |
| *Disorganization a = 0.882* | Derailment  Tangentiality  Incoherence | SAPS  SAPS  SAPS |
| *Disordered Relating a = 0.892* | Recreational interests and activities  Sexual activity  Ability to feel intimacy and closeness  Relationships with friends and peers | SANS  SANS  SANS  SANS |
| *Bizarre Delusions a = 0.715* | Delusions of being controlled  Delusions of mind reading  Thought broadcasting  Thought insertion  Thought withdrawal | SAPS  SAPS  SAPS  SAPS  SAPS |
| *Auditory Hallucinations a = 0.683* | Auditory hallucinations  Voices conversing  Voices commenting | SAPS  SAPS  SAPS |
